# Metformin's Effects on Apoptosis of Esophageal Carcinoma Cells and Normal Esophageal Epithelial Cells: An In Vitro Comparative Study

**DOI:** 10.1155/2020/1068671

**Published:** 2020-03-18

**Authors:** Jianwei Peng, Xubin Jing, Jialing Wu, Danmian Hong, Xi Hu, Qinjia Wang, Hui Hu, Xianbin Cai

**Affiliations:** ^1^Department of Gastroenterology, The First Affiliated Hospital of Shantou University Medical College, Shantou, Guangdong 515041, China; ^2^Department of Gastroenterology, Jieyang People's Hospital, Jieyang, Guangdong 522010, China

## Abstract

The effect of metformin on human esophageal normal and carcinoma cells remains poorly understood. We aim to investigate the different antiproliferation effects and underlying distinct molecular mechanisms between these two types of cells. Human esophageal squamous cell carcinoma cell line, EC109, and normal esophageal epithelial cell line, HEEC, were used in the experiment. The cell survival rate was determined by cell counting kit-8 (CCK-8). Cell apoptosis was analyzed by flow cytometry. The mRNA and protein levels of signal transducer and activator of transcription 3 (Stat3) were detected by real-time quantitative PCR and western blot. Interleukin-6 (IL-6) was added to activate Stat3. The level of intracellular reactive oxygen species (ROS) was assessed by a DCFH-DA fluorescent probe. Metformin had more significant inhibitory effects on cell proliferation in EC109 cells than HEECs. Metformin induced apoptosis of EC109 cells in a dose-dependent manner instead of HEECs. The expression of Stat3 in both mRNA and protein levels was higher in EC109 cells than HEECs. Further study revealed that metformin may attenuate the phosphorylation of the Stat3 and the Bcl-2 expression, which was restored by IL-6 partly in EC109 cells but not HEECs. On the contrary, metformin increased the level of ROS in both the cell lines, but this intracellular ROS variation had no effect on apoptosis. Metformin has different functional roles on the apoptosis in esophageal carcinoma cells and normal esophageal cells. Therefore, the Stat3/Bcl-2 pathway-mediated apoptosis underlies the cell-type-specific drug sensitivity, suggesting metformin possesses a therapeutic activity and selectivity on esophageal cancer.

## 1. Introduction

Esophageal cancer (EC) is one of the most common malignant tumors. It was estimated that there were 455800 cases of EC and 400200 deaths in one year. And esophageal squamous cell carcinoma (ESCC) is the predominant subtype of EC and highly prevalent in China, especially the north-central region and ChaoShan region [1, 2]. Currently, comprehensive therapy to combine surgery with radio-chemotherapy is the main therapeutic strategy for this fatal disease. However, the serious side effects limit the application of traditional radio-chemotherapy. Although the therapeutic strategy improves, the five-year survival rate for EC is no more than 20% [3]. It is necessary to look for new antitumor drugs with minimal damage to normal cells.

Recent studies have shown that metformin, a widely used antidiabetic drug, exhibits anticancer effects. It has no significant hypoglycemic effect on normal subjects and no risk of hypoglycemia when used alone [4]. In 2005, a case-control study showed that the long-term usage of metformin significantly reduced the risk of cancer in the diabetic population [5]. Thereafter, emerged epidemiological studies from different regions supported this conclusion [6–8]. Moreover, metformin has been identified to inhibit various human cancer cells in vitro and in vivo, including breast cancer [9], ovarian cancer [10], and pancreatic cancer [11].

Nevertheless, the molecular mechanisms of metformin's antitumor effect were discovered but still remained unclear. Signal transducer and activator of transcription 3 (Stat3) is a cytoplasmic transcription factor that can be activated by various factors and cytokines, such as IL-6 [12]. Stat3 is present in an inactive state in the normal cells, while it is in a continuously active state in a variety of cancer cells [13, 14]. Studies have also shown that abnormal activation of Stat3 was associated with poor prognosis in cancer patients [15, 16]. Inhibition of Stat3 could block the abnormal signal transduction and biological effects of target genes, so Stat3 may be a therapeutic target in treatment of tumors. It has been reported that metformin could inhibit proliferation and induce apoptosis of breast cancer cells with Stat3 as a target [17].

Reactive oxygen species (ROS) are constantly generated and eliminated in the biological system and play important roles in various pathophysiological processes [18]. Our previous studies have shown that intracellular ROS contributed to cisplatin-induced apoptosis in ESCC [19]. Studies have found that metformin could induce apoptosis of tumor cells by increasing the level of ROS [9]. On the contrary, metformin was reported to decrease ROS accumulation in cells, reducing the DNA damage and mutation of normal cells [20].

However, few studies focus on exploring the distinction of metformin on human esophageal normal epithelial cells and carcinoma cells. In this study, we evaluated the antiapoptosis effects of metformin on esophageal carcinoma cells and normal epithelial cells in the in vitro model and investigated the role of Stat3 signaling and intracellular ROS.

## 2. Materials and Methods

### 2.1. Cell Lines, Medium, and Cell Culture

The human ESCC tumor cell line EC109 and normal esophageal epithelial cell line HEEC were obtained from the Type Culture Collection of the Chinese Academy of Sciences (Shanghai, China). All cells were cultured in 10% fetal bovine serum (FBS) with Dulbecco's modified Eagle's medium (DMEM) (both from Gibco, Thermo Fisher Scientific, Inc., Waltham, MA, USA) without antibiotics at 37°C with 5% CO_2_.

### 2.2. CCK-8 Assay

Cell viability was assessed by CCK-8 (Dojindo, Kumamoto, Japan). The cells were seeded at 5 × 10^3^ cells/well in 96-well plates for 24 h. Then, the medium was exchanged for the fresh culture medium with metformin (Sigma-Aldrich, St. Louis, MO, USA) in indicated concentrations (0, 2.5, 5.0, 10, and 20 mM) for 24, 48, and 72 h. The absorbance was measured at 450 nm by a microplate reader. All determinations were confirmed using three independent replicate experiments.

### 2.3. Analysis of Apoptosis

The cell apoptosis detection kit Annexin V-FITC/PI Kit was purchased from 4A Biotech Co., Ltd. (Beijing, China). The cells were seeded at 5 × 10^4^ cells/well in 12-well plates in triplicate for 24 h. The medium was removed and replaced with the fresh culture medium containing metformin in various concentrations (0, 2.5, 5.0, 10, and 20 mM) for 24 h. The apoptotic cells were examined by flow cytometry (Beckman Coulter, Inc., Brea, CA) of the cells labeled with Annexin V-FITC and PI following the manufacturer's instructions.

### 2.4. Real-Time PCR Analysis

Total RNA was extracted from EC109 cells and HEECs using Trizol (Invitrogen). The cDNA was synthesized from mRNA with a PrimeScript Kit (TAKARA, Toyobo, Osaka, Japan). Gene transcription levels were accessed using real-time quantitative PCR with SYBR Green PCR Master Mix (TAKARA). At the same time, GAPDH was measured as an endogenous control. All the samples were quantified three times. The mRNA levels were expressed using the 2^−ΔΔCT^ method. The PCR was performed with a two-step initial denaturation at 95°C for 30 sec and then 40 cycles of 95°C for 5 sec and 60°C for 30 sec. Primer sequences used are listed as follows:

  Stat3: forward: AATAATTTGCACTCCTCCTCCA; reverse: GTTAAGAACCACCCAGCTTGTC
  GAPDH: forward: GAGCCAAAAGGGTCATCATCTC; reverse: AAAGGTGGAGGAGTGGGTGTC

### 2.5. Western Blot Analysis

The EC109 cells and HEECs were harvested and lysed in the radio-immunoprecipitation assay (RIPA) buffer (Thermo Fisher, Rockford, IL, USA) containing protease and phosphatase inhibitors (1 *μ*l), NaF (1 mM, 1 *μ*l), sodium orthovanadate (1 mM, 1 *μ*l), and PMSF (1 mM, 1 *μ*l), homogenized, and then centrifuged (12000 ×g at 4°C for 10 min) Protein concentration was measured with Bicinchoninic Acid Protein Kit (Pierce, IL, USA). A total of 30 *μ*g protein was isolated from each sample by SDS-PAGE gel electrophoresis and transferred onto a PVDF membrane (Millipore, Shanghai). The membranes were then blocked in the TBS/Tween 20 (TBST) buffer containing 10% nonfat milk powder for 2 h at room temperature and subsequently incubated with primary antibodies overnight at 4°C. Anti-phospho-Stat3, anti-Stat3, and mouse anti-GAPDH antibodies were from Cell Signaling Technologies (Beverly, MA, USA). Bcl-2 antibodies were from Santa Cruz (Santa Cruz, CA, USA). The rabbit anti-GAPDH antibody was from Abcam. After probing with secondary antibodies for 1 h at room temperature, the membranes were washed and developed with the ECL (enhanced chemiluminescent) detection system (Bio-Rad). GAPDH was used as loading controls.

### 2.6. Assessment of ROS Levels

The cells were plated at 5 × 10^4^ cells/well on 12-well plates before treatment. The medium was then replaced with the fresh medium containing various concentrations of metformin (0, 2.5, 5.0, 10, and 20 mM). Then, the cells were washed three times and assessed for fluorescence intensity by using fluorescent microscopy. Similarly, the cells were measured by flow cytometry (Beckman Coulter, Inc., Brea, CA), and the number of cells was 1 × 10^4^, excitation wavelength 488 nm, and emission wavelength 525 nm.

### 2.7. Statistical Analysis

All data were presented as mean ± SD. GraphPad Prism 5.0 was used for statistical analysis. Statistical analysis of the results was performed using Student's *t*-test for only two groups or using one-way analysis of variance (ANOVA) when there were more than two groups. *P* < 0.05 was considered statistically significant, and *P* < 0.01 was considered highly statistically significant.

## 3. Results

### 3.1. Metformin Inhibited Growth of ESCC Cells and Normal Esophageal Epithelial Cells

**Figure 1:**
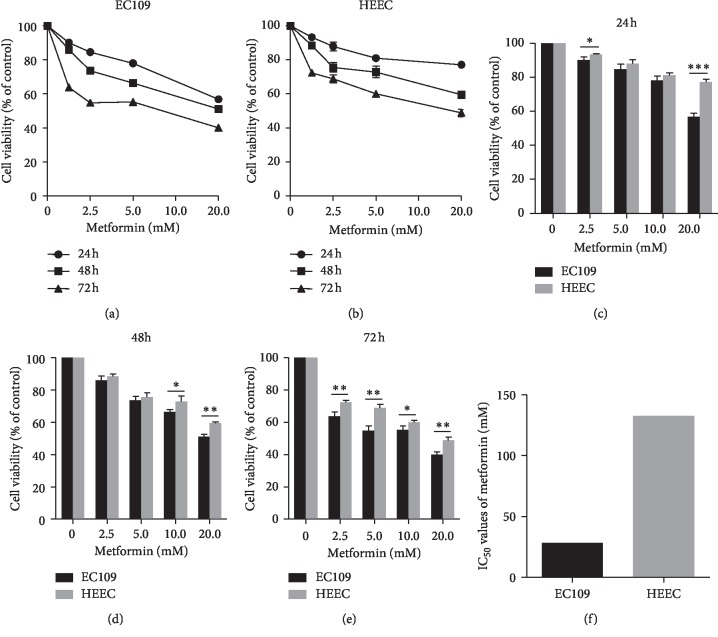
Metformin inhibited the viability of ESCC cells and normal esophageal epithelial cells. EC109 cells (a) and HEECs (b) were treated with metformin at different concentrations for 24 h (c), 48 h (d), and 72 h (e). Cell viability was evaluated by CCK-8. (f) IC_50_ of metformin in EC109 cells and HEECs at 24 h. IC_50_: half maximal inhibitory concentration. Data were presented as mean ± SD (*n* = 3). ^*∗*^*P* < 0.05;^*∗∗*^*P* < 0.01;^*∗∗∗*^*P* < 0.001.

To explore the effect of metformin on the growth of ESCC cells and normal esophageal epithelial cells, EC109 cells and HEECs were treated with indicated concentrations of metformin for 24 h, 48 h, and 72 h. The cell viability was determined by the CCK-8 assay. The results showed that metformin inhibited the growth of EC109 cells and HEECs in dose- and time-dependent ways (Figures 1(a)–1(e)). The half maximal inhibitory concentration (IC_50_) of metformin on EC109 cells and HEECs was 28.08 and 132.7 mM, respectively (Figure 1(f)). This result shows that metformin inhibits the growth of EC109 cells more than HEECs.

### 3.2. Metformin Induced Apoptosis in ESCC Cells but Not in Normal Esophageal Epithelial Cells

**Figure 2:**
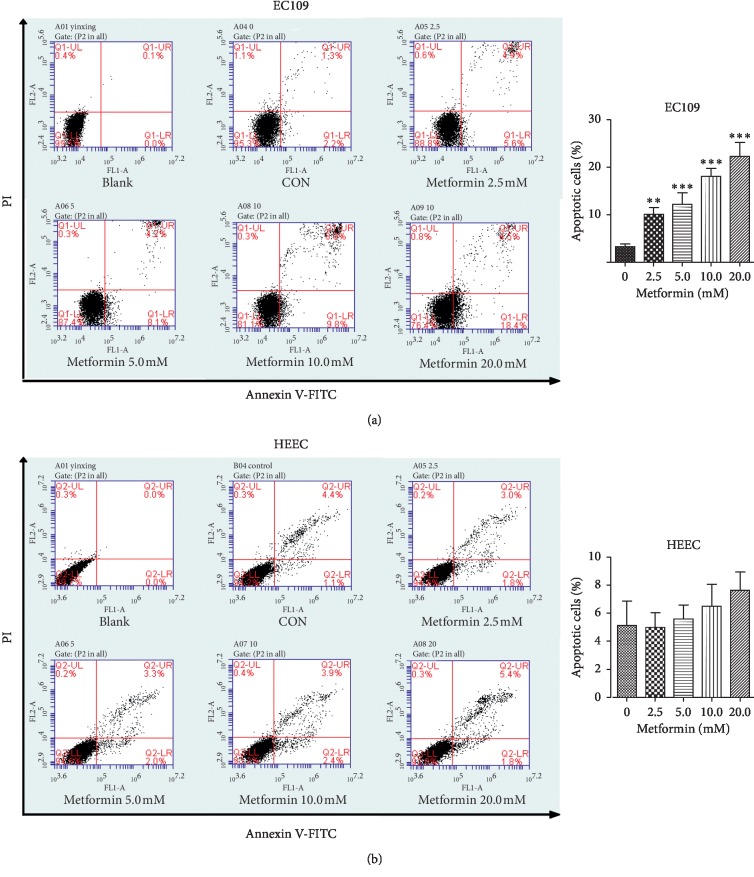
Metformin induced apoptosis in ESCC cells. EC109 cells and HEECs were treated with metformin at different concentrations for 24 h. The apoptotic index (%) of EC109 cells (a) and HEECs (b) was determined by flow cytometry analysis using Annexin V-FITC/PI double staining. Data were presented as mean ± SD (*n* = 3). ^*∗*^*P* < 0.05,^*∗∗*^*P* < 0.01,  *and* ^*∗∗∗*^*P* < 0.001 versus the corresponding control (0 mM). CON: control.

As the metformin suppressed the viability of both cell lines, to further evaluate whether metformin causes cell death, Annexin V-FITC/PI flow cytometry was used to indicate apoptosis of cells. Metformin increased the percentage of apoptotic cells in EC109 cells in a range from 0 mM to 20 mM (Figure 2(a)), which did not appear in HEECs (Figure 2(b)). Together, these data indicate that metformin selectively induces apoptosis in ESCC cells without affecting the normal esophageal epithelial cells.

### 3.3. Expression of Stat3 in ESCC Cells and Normal Esophageal Epithelial Cells

It is well known that Stat3 is constitutively activated in numerous cancer types, and metformin suppresses the Stat3 activation in lung cancer and ESCC cells [21, 22]. We therefore detected the expression of Stat3 mRNA and protein levels with real-time PCR technology and western blot. As expected, the basal level of Stat3 expression and the Stat3 phosphorylation in EC109 cells were significantly higher than those in HEECs ([Supplementary-material supplementary-material-1]), suggesting that Stat3 variation is involved in the difference of metformin-induced apoptotic cell death in EC109 cells and HEECs.

### 3.4. Metformin Downregulated the Phosphorylation of Stat3/Bcl-2 Pathway in ESCC Cells instead of Normal Esophageal Epithelial Cells

**Figure 3:**
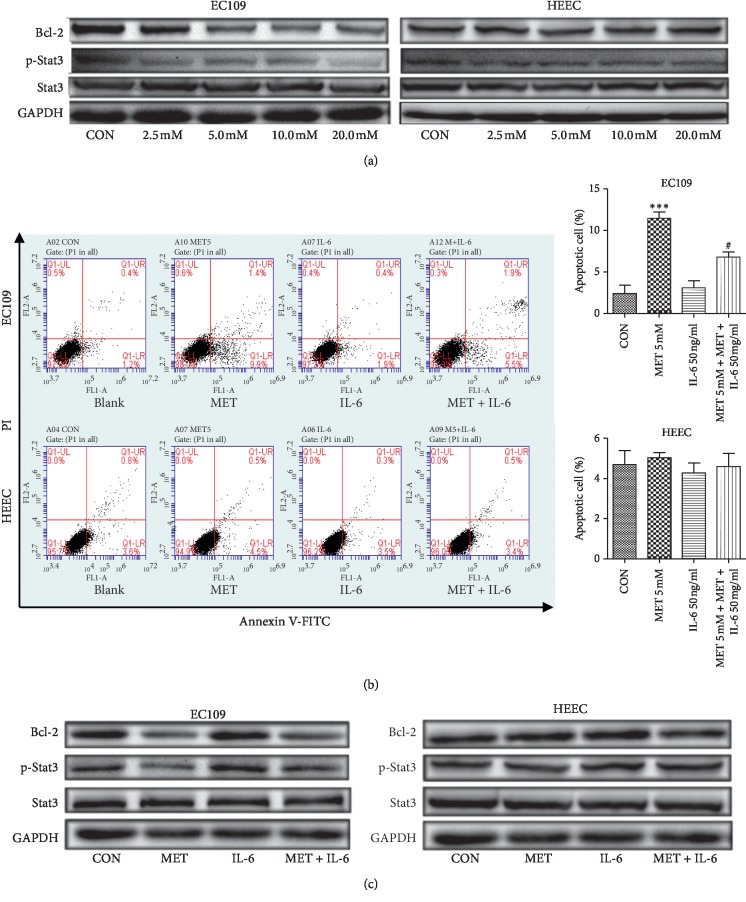
Effect of metformin on the phosphorylation level of Stat3 and the expression of Bcl-2 in ESCC cells and normal esophageal epithelial cells. (a) Levels of Stat3 and Bcl-2 were determined by western blot in EC109 cells and HEECs after treatment with metformin at different concentrations. (b) Effects of metformin treatment with or without IL-6 on cell apoptosis in EC109 cells and HEECs. The apoptotic index (%) of EC109 cells and HEECs was determined by Annexin V-FITC/PI flow cytometry. (c) Phosphorylated Stat3 and Bcl-2 were assayed by western blot in EC109 cells and HEECs. GAPDH was probed as the loading control. CON: control; MET: metformin. Data were presented as mean ± SD (*n* = 3). ^*∗*^*P* < 0.05,^*∗∗*^*P* < 0.01,  *and* ^*∗∗∗*^*P* < 0.001 versus the control. ^#^*P* < 0.05 versus the MET 5 mM group.

Aberrant Stat3 activation stimulates tumor cell proliferation through inhibition of apoptosis, a function mediated by upregulation of the antiapoptotic gene Bcl-2 [23]. In order to investigate the influence of metformin on Stat3 and downstream Bcl-2 in EC109 cells and HEECs, two cell lines were treated with 0, 2.5, 5.0, 10.0, and 20.0 mM of metformin for 24 h, respectively. The level of proteins was assayed by western blot. As a result, metformin repressed the phosphorylation of Stat3 and expression of Bcl-2 in a dose-dependent manner. In contrast, these changes were not found in HEECs (Figure 3(a)). To further validate the negative regulation of Stat3 signaling in apoptosis in metformin-treated cells, we added IL-6 to activate Stat3. Metformin-mediated apoptosis of EC109 cells was reversed after treatment with IL-6. Metformin and IL-6 had no obvious effect on HEECs (Figure 3(b)). Correspondingly, IL-6 abrogated the downregulation of p-Stat3 and Bcl-2 by metformin in EC109 cells. Although IL-6 activated Stat3 in HEECs, Bcl-2 remained stable (Figure 3(c)). These findings indicated that Stat3/Bcl-2 signaling participated in metformin-induced apoptosis of ESCC cells, but not in the normal esophageal epithelial cells.

### 3.5. The Relationship between Metformin and ROS Accumulation/Apoptosis in ESCC Cells and Normal Esophageal Epithelial Cells

**Figure 4:**
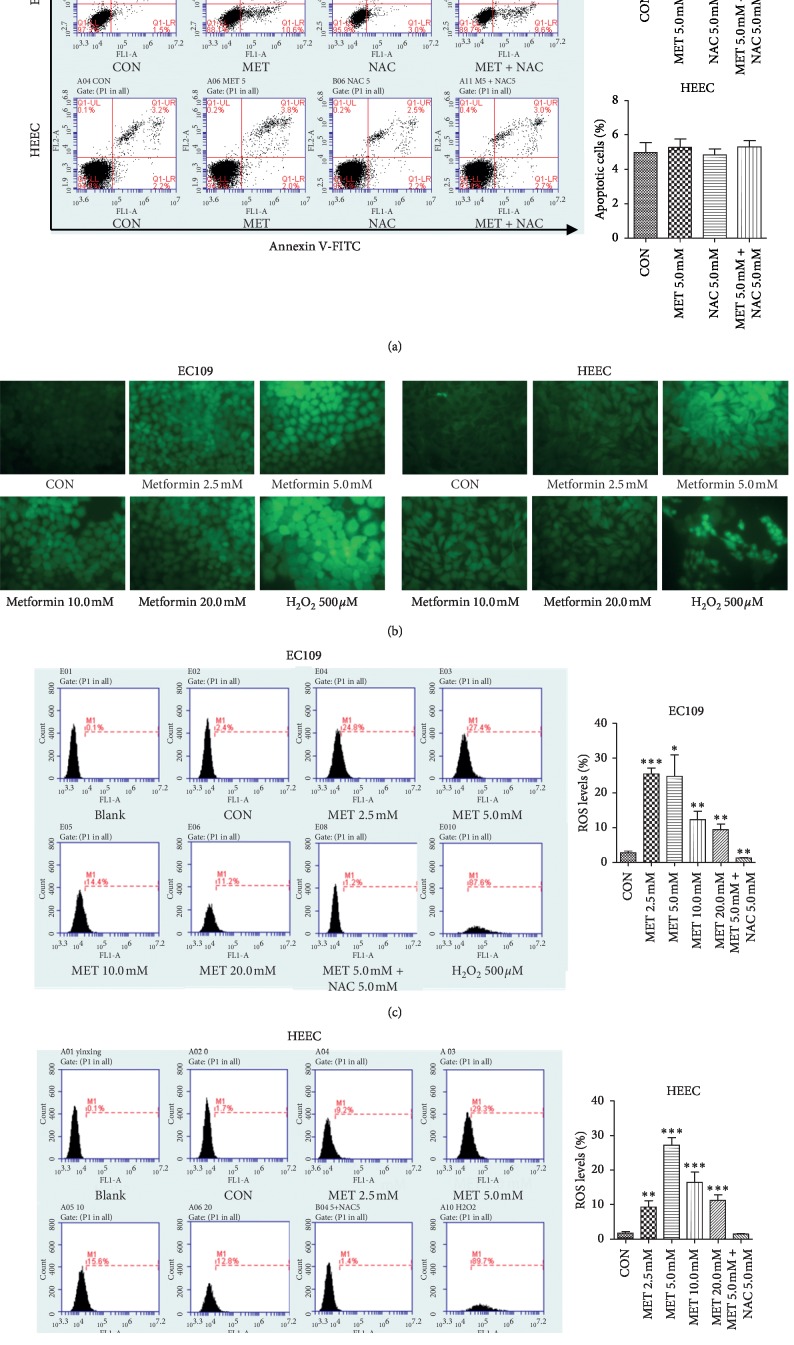
Relationship between metformin-induced cell apoptosis and the level of intracellular ROS in EC109 cells and HEECs. (a) The apoptotic index (%) of EC109 cells and HEECs was determined by flow cytometry analysis using Annexin V-FITC/PI double staining upon treatment with metformin and/or NAC. (b) Cells were imaged on a fluorescence microscope after treatment with the indicated concentration of metformin or H_2_O_2_ for 24 h (magnification ×400). (c, d) Flow cytometry was used to detect the intracellular ROS levels after the indicated concentration of metformin for 24 h and preincubation with 5.0 mM NAC for 2 h before exposure to 5.0 mM metformin for 24 h. Data were presented as mean ± SD (*n* = 3). ^*∗*^*P* < 0.05,^*∗∗*^*P* < 0.01,  *and* ^*∗∗∗*^*P* < 0.001 versus the corresponding control. CON: control; MET: metformin.

Metformin can also induce the generation of ROS and disrupt the mitochondrial membrane potential, leading to apoptosis in breast cancer cells [24]. Therefore, the intracellular ROS levels were assayed with the DCFH-DA fluorescent probe and flow cytometry. Green fluorescence intensity in EC109 cells and HEECs was elevated after treatment with different concentrations of metformin (Figure 4(b)). Besides this, it was suggested that stimulation with metformin (5 mM) generated ROS with a peak at 24 h. As expected, addition with NAC, an oxygen scavenger, weakened prooxidant effects of metformin on EC109 cells and HEECs (Figures 4(b)–4(d)). Then, the apoptosis of cells was evaluated by flow cytometry analysis by Annexin V-FITC/PI double staining. However, NAC could not rescue the apoptosis induced by metformin in ESCC cells. In addition, no difference was observed between the HEECs treated with or without metformin and/or NAC on cell apoptosis (Figure 4(a)). In sum, the intracellular ROS accumulation has no relation with the metformin proapoptotic effect in both the cell lines.

## 4. Discussion

In this study, we investigated the effect of metformin on ESCC cells and normal esophageal epithelial cells and the underlying molecular mechanisms. Here, we found that metformin inhibited the proliferation in ESCC cells more than the normal esophageal epithelial cells, as well as the apoptosis rate. And only the Stat3 signaling pathway but not the ROS signaling may underline the different effect of metformin on the EC109 cells and HEECs. These indicated that metformin is safe and promising for the prevention and cure of esophageal carcinoma.

Metformin has been shown to have the inhibitory effect on esophageal carcinoma cells [25, 26]. However, few studies focused on the relationship between metformin and normal esophageal cells. Our previous experiment had confirmed the inhibitory effect of metformin on the proliferation of esophageal carcinoma cells. Based on this, we found that metformin had less antiproliferation and no antiapoptosis effects on normal esophageal epithelial cells compared with ESCC cells (Figures 1 and 2). The significantly high IC_50_ in HEECs indicated that HEECs have a great tolerance to metformin than EC109 cells. In other words, the therapeutic dosage of metformin on ESCC is harmless to the normal esophageal tissue. Another report also confirmed metformin marginally reduced the viability of NE3 cells, a kind of esophageal epithelial cell line, which was in line with our result [22].

To our surprise, we found that the Stat3 expression level and activation in EC109 cells were significantly higher than those in HEECs, provoking our further investigation on the role of Stat3 in these two types of cell lines. Stat3 is a meeting point for many cytokines, growth factors, oncogenes, and inflammatory signaling pathways [27]. Stat3 is closely related to the tumor initiation and development especially in ESCC, which is associated with persistent inflammation. The prosurvival protein Bcl-2 is a downstream target gene of Stat3 and plays an important role in apoptosis regulation. Studies showed that Stat3 mainly triggers apoptosis by regulating the expression of Bcl-2 [28, 29]. The activation of Stat3 in normal cells is fast and transient, but it is continuously activated in tumor cells. Overexpression of Stat3 has been reported in human esophageal carcinoma, and it is related to malignancy and prognosis of the tumor [30]. Similarly, we found that ESCC cells and HEECs displayed the different level of Stat3 activation. Meanwhile, metformin inhibited the Stat3 phosphorylation and downregulated the Bcl-2 expression accompanied by induced apoptosis of ESCC cells. In contrast, metformin had no significant effect on the Stat3 activation, Bcl-2 expression, and apoptosis in normal esophageal epithelial cells. Accordingly, we speculated that the difference of Stat3 expression and phosphorylation may be a cause why metformin acted differently on the two cells. It was found that Stat3 knockdown intensified Bcl-2 repression by metformin [22]. Likewise, in our study, metformin-induced antitumor effect and the repression of Stat3 activation and Bcl-2 expression could be partly blocked by IL-6, a Stat3 activator. Interestingly, we observed that IL-6 could phosphorylate Stat3 but does not impact the apoptosis and Bcl-2 expression in normal esophageal epithelial cells exposed to metformin. This suggested that metformin induced apoptosis of esophageal carcinoma cells partly regulated by the Stat3/Bcl-2 pathway, which may have little function on normal esophageal epithelial cells.

Growing evidence has demonstrated that the level of ROS and the antioxidant capacity displayed distinct differences between tumor cells and normal cells [31]. Metformin was reported to reduce ROS [11] production or act as a prooxidant [32, 33]. We found that the level of ROS was elevated in ESCC cells in a dose-dependent manner with the treatment of metformin, which was inconsistent with the apoptosis rate (Figure 4). In contrast, Stat3 activation induced by metformin is in parallel with the apoptosis rate. It is an established fact that ROS can trigger the intrinsic (or mitochondrial) pathway to regulate the cell apoptosis [34]. Surprisingly, our results showed that NAC, an oxygen scavenger, can reduce the production of ROS in EC109 cells when exposed to metformin, but it could not reverse the metformin-mediated apoptosis of EC109 cells. However, Li et al. reported that metformin combined with cisplatin could synergistically increase the level of ROS and induce apoptosis in ESCC cells (Eca109 and KYSE30) [33]. This suggested that the accumulation of ROS mediated by metformin alone is not enough to induce cell apoptosis without other agents, such as cisplatin, or the metformin-induced ROS could not affect the apoptosis of EC109 cells at all. Therefore, the multifaceted roles of ROS and the complexity of the ROS homeostasis system for different cells should be considered. Further studies are necessary to make this clear. In addition, metformin also increases the ROS level in HEECs as in EC109 cells. But the HEEC's apoptosis was not exacerbated after exposed to metformin with or without NAC. From these data, we can conclude that the ROS pathway may not be involved in the metformin antitumor effect, which may not account for the different effect of metformin on the EC109 cells and HEECs.

Overall, this study demonstrates that ESCC cells and normal esophageal epithelial cells had different sensitivity to metformin treatment. The potential molecular mechanism may involve in the Stat3 signaling pathway but not in ROS production. Our findings provide comprehensive insights into the therapeutic application of metformin with minimum side effects and high efficacy on esophageal cancer.

## Data Availability

The datasets used and/or analyzed during the current study are available from the corresponding author on reasonable request.

## Supplementary Materials

Supplementary Materials Fig. S1: expression of Stat3 in mRNA levels and protein levels between ESCC cells and normal esophageal epithelial cells. (A) mRNA levels of Stat3 between EC109 cells and HEECs expressed as the ratio of the expression in EC109 cells. (B) Protein expression of total and phosphorylated Stat3 in EC109 cells and HEECs. GAPDH was probed as the loading control. Data were presented as mean ± SD (n=3). ^*∗∗*^*P* < 0.01, compared to the EC109 group.
