# Supplementary material for: Metformin's Effects on Apoptosis of Esophageal Carcinoma Cells and Normal Esophageal Epithelial Cells: An In Vitro Comparative Study
Source: Biomed Res Int. 2020 Mar 18;2020:1068671. doi: 10.1155/2020/1068671 (PMC7104266; doi:10.1155/2020/1068671)
Supplement: Supplementary Materials — Fig. S1: expression of Stat3 in mRNA levels and protein levels between ESCC cells and normal esophageal epithelial cells. (A) mRNA levels of Stat3 between EC109 cells and HEECs expressed as the ratio of the expression in EC109 cells. (B) Protein expression of total and phosphorylated Stat3 in EC109 cells and HEECs. GAPDH was probed as the loading control. Data were presented as mean ± SD (n=3). ∗∗P < 0.01, compared to the EC109 group. [file 1068671.f1.docx]

**Supplementary Materials**

**Figure S1**

**
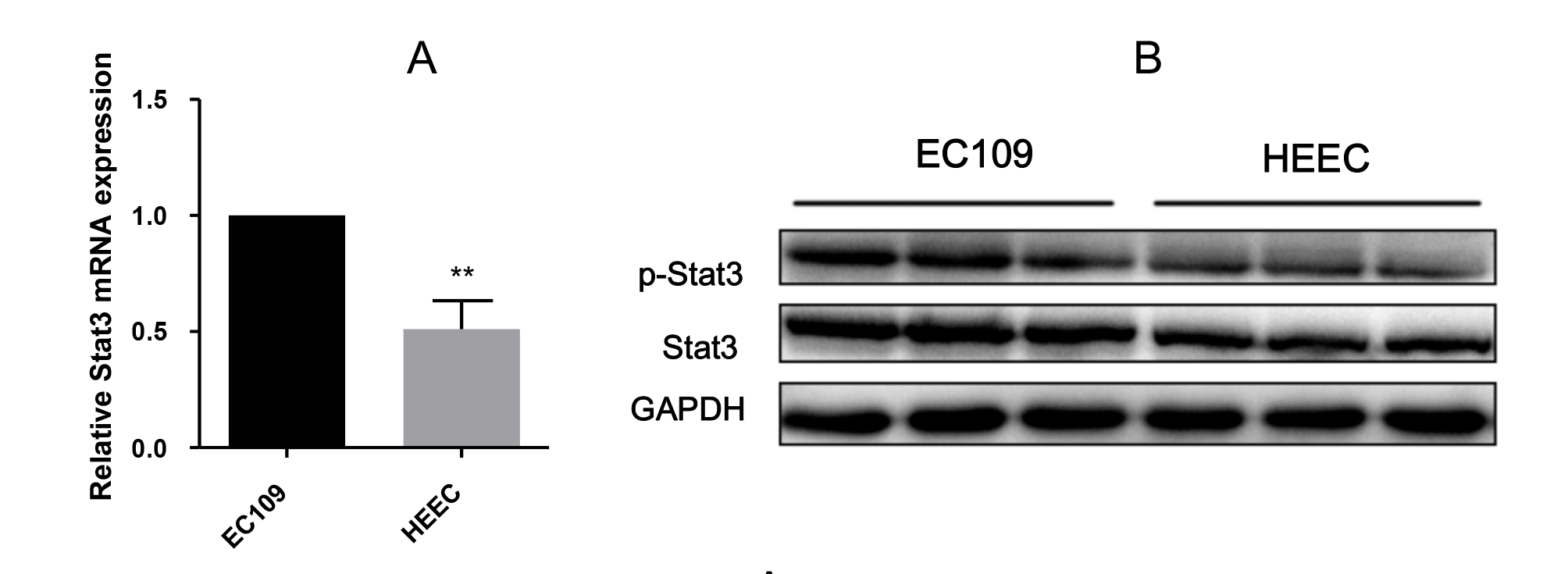
**

**Fig. S1.** The expression of Stat3 in mRNA levels and protein levels between ESCCs and normal esophageal epithelial cells. **A.** The mRNA levels of Stat3 between EC109 and HEEC cells, was expressed as the ratio of the expression in EC109. **B.** The protein expression of total and phosphorylated Stat3 in EC109 and HEEC cells. GAPDH was probed as the loading control. Data was presented as mean ± SD (n=3). ***P*<0.01, compared to EC109 group.
